# Screening Hepatotoxic Components in *Euodia rutaecarpa* by UHPLC-QTOF/MS Based on the Spectrum-Toxicity Relationship

**DOI:** 10.3390/molecules22081264

**Published:** 2017-07-27

**Authors:** Jian Liang, Yang Chen, Gang Ren, Wei Dong, Min Shi, Li Xiong, Jiankang Li, Jiahao Dong, Fei Li, Jinbin Yuan

**Affiliations:** 1 Key Lab of Modern Preparations of TCM, Jiangxi University of Traditional Chinese Medicine, Nanchang 330004, China; ocean719@163.com (J.L.); chenyang_0307@126.com (Y.C.); firmblue@163.com (G.R.); sober96@foxmail.com (W.D.); flea_sh@126.com (M.S.); lijiankang913@163.com (J.L.); dongjh233@163.com (J.D.); lfzhy0408@163.com (F.L.); 2 Jiangxi Province Center for Disease Control and Prevention, Nanchang 330004, China; xionglibb@163.com

**Keywords:** *Euodia rutaecarpa*, spectrum-toxicity relationship, hepatotoxicity, UHPLC-Q-TOF/MS, OPLS

## Abstract

*Euodia rutaecarpa* is a common traditional Chinese medicine (TCM) in clinical practice, having the ability to suppress pain and cease coughing; however, with the increasing reports showing that it is toxic, particularly hepatotoxic, the concerns raised by what cause its toxicity is growing. In the current study, an analysis method based on the spectrum effect has been employed to screen the major hepatotoxic components in *Euodia rutaecarpa* so that the toxic material’s basis would be elucidated. A fingerprinting method of the *Euodia rutaecarpa* extracts (which were petroleum ether, chloroform, ethyl acetate, *n*-butanol, and water) using ultra-high-performance liquid chromatography coupled with quadrupole time-of-flight mass spectrometer (UHPLC-QTOF/MS) has been developed. Orthogonal partial least squares (OPLS) was used to establish the spectrum-toxicity relationship with the levels of alanine aminotransferase (ALT) and aspartate aminotransferase (AST) in mice serum as evaluation indices for liver injury. The UHPLC-MS fingerprint was established and the OPLS analytical results suggested that coniferin, 1-methyl-2-undecyl-4(1*H*)-quinolone, 1-methyl-2-[(6*Z*,9*Z*,12*E*)-pentadeca triene]-4(1*H*)-quinolone, evocarpine, 1-methyl-2-[(*Z*)-7-tridecenyl]-4(1*H*)-quinolone, dihydroevocarpine, and 1-methyl-2-tetradecy-4-(1*H*)-quinolone probably associated with the hepatotoxicity of *Euodia rutaecarpa*. This paper offered considerable methods and insight for the fundamental research of the toxic material basis of similar toxic TCMs.

## 1. Introduction

Traditionally Chinese medicine (TCM) has been considered safer, or even harmless, for decades in peoples’ minds in comparison with western medicine, which is also called chemical medicine in China; nevertheless, increasing studies have found the reality to be otherwise. German scientist Teschke and his team compiled worldwide cases of liver injury by herbal TCM through the PubMed database, and they identified reported hepatotoxicity cases including *Euodia rutaecarpa* in 77 relevant publications with 57 different herbs and herbal mixtures of TCM [1]. German and French scientists also conducted similar data mining using PubMed database [2]. They found out that the herb-induced liver injury rate and similarly to drugs can be caused by an unpredictable idiosyncratic or a predictable intrinsic reaction, and the suspected cases deserve special clinical and regulatory attention. *Euodia rutaecarpa* is one of those toxic herbal TCMs. It is the dry and mature fruit of *Euodia rutaecarpa* (Juss.) Benth., *E. rutaecarpa* (Juss.) Benth. var. *officinalis* (Dode) Huang or *Euodia rutaecarpa* (Juss.) Benth. var. *bodinier* (Dode) Huang [3]. It has been widely used for thousands of years in Southeast Asian countries, including China, Japan, and Korea. *Euodia rutaecarpa*, as a traditional ethnodrug, has a definite curative effect. The efficacy of ER has been well described in the Chinese materia medica and Chinese pharmacopoeia, and has been confirmed by modern pharmacology. As a common TCM in clinical practice, it has the ability to suppress pain and cease coughing, hence, it is frequently used to treat dizziness, headache, costalgia, emesis, diarrhea, and other diseases. Modern pharmacology studies indicate that *Euodia rutaecarpa* has many pharmacological activities such as anti-inflammatory, cardioprotective, and hypotensive effects [4]. Like any other medical plant, there are major constituents in *Euodia rutaecarpa* that fall into various chemical categories, including alkaloids, limonins, terpenes, flavonoids, coumarins, steroids, polysaccharides, and so on [5]. Both previous literature and current the Chinese Pharmacopeia record that it is mildly toxic or toxic. In recent years, there are growing clinical reports on its adverse effects—in particular, hepatotoxicity [1,6,7]—which has led to a public concern about what these toxic constituents are exactly. Earlier, Xiuwei Yang’s team worried about these toxic and side effects and, therefore, studied the acute and genetic toxicity of aqueous and 70% alcohol extracts systematically, with no notable toxicity being found [8]. Huang et al. observed and investigated the toxic and side effects that were caused by *Euodia rutaecarpa* under therapeutic dose. The findings indicated that its water extracts were able to reduce the frequency of writhing times of mice induced by acetic acid and dramatically raise the pain threshold induced by a hot plate in a good dose-dependent manner. They also found that the ALT and AST levels in serum and liver tissue increased greatly, as did the liver-to-body ratio; these changes become more severe gradually when the dose was increased, showing notable differences in comparison with the control group in a certain dose-dependent manner. Beyond that, they also ran a series of tests on acute toxicity of different constituents, hepatotoxicity of volatile oil and water extracts [9,10,11,12], and both acute toxicity and hepatotoxicity of ethanol extract [13,14]; they verified that volatile oil and alcohol extracts are the main toxic fractions, and discovered an interesting fact that there are close connections between its efficacy and toxicity of different extracts. The duality of efficacy and toxicity means its efficacy and toxicity go hand in hand, and these two effects are dose-connected. Until today, there have only been a small number of reports on the toxic material of *Euodia rutaecarpa*, hence, it is necessary to further elucidate the toxic material bases to provide scientific evidence for quality control and safe usage.

Due to the vast constituents in TCM, it is a challenging task to find the very materials that are responsible for its toxicity [15], and a fingerprinting technique is a good approach to solve this problem. It is one of the key techniques of modern TCM research, and the spectrum-effect relationship is the higher stage of fingerprinting research. The analysis on fingerprint and pharmacologic efficacy not only leads to the revelation of their correlation in between, but can also help with determining the active compounds [16]. Spectrum-effect relationship research has been widely applied to many fields, such as active compounds of TCM [17], prescription composition, drug processing mechanism [18], prediction of pharmacologic activity [19,20,21], and so on. The studies on relevant areas are in increasing need of the assistance of complex system modeling, and OPLS is one of them. OPLS is an improved partial least squares method being capable of eliminating the uncorrelated information to variable *Y* from the prediction matrix and improving the explanatory ability and accuracy of the model [17]. Combining the basic functions of multi-variable linear regression analysis, canonical correlation analysis, and principal component analysis, it could find the correlation between chromatographic peaks and toxicity and how strong the correlation is in an accurate manner. In addition, OPLS makes the best use of obtained data and is a good model that can accurately predict new training sets and be easily interpreted. It is, therefore, a very effective approach to study the spectrum-effect relationship owing to these merits mentioned above [18].

The UHPLC-MS technique was employed in this work to develop the characteristic fingerprint of *Euodia rutaecarpa*; serum levels of ALT and AST served as the evaluation indices for liver injury. Turning the spectrum-effect relationship into a spectrum-toxicity relationship study, the major toxic components of *Euodia rutaecarpa* have been carefully explored by OPLS in this paper. The aim of this study was to elucidate and screen potential toxic components for further study, which may also lead to similar work in other toxic TCMs.

## 2. Results and Discussion

### 2.1. Assignment of Major Components

**Figure 1:**
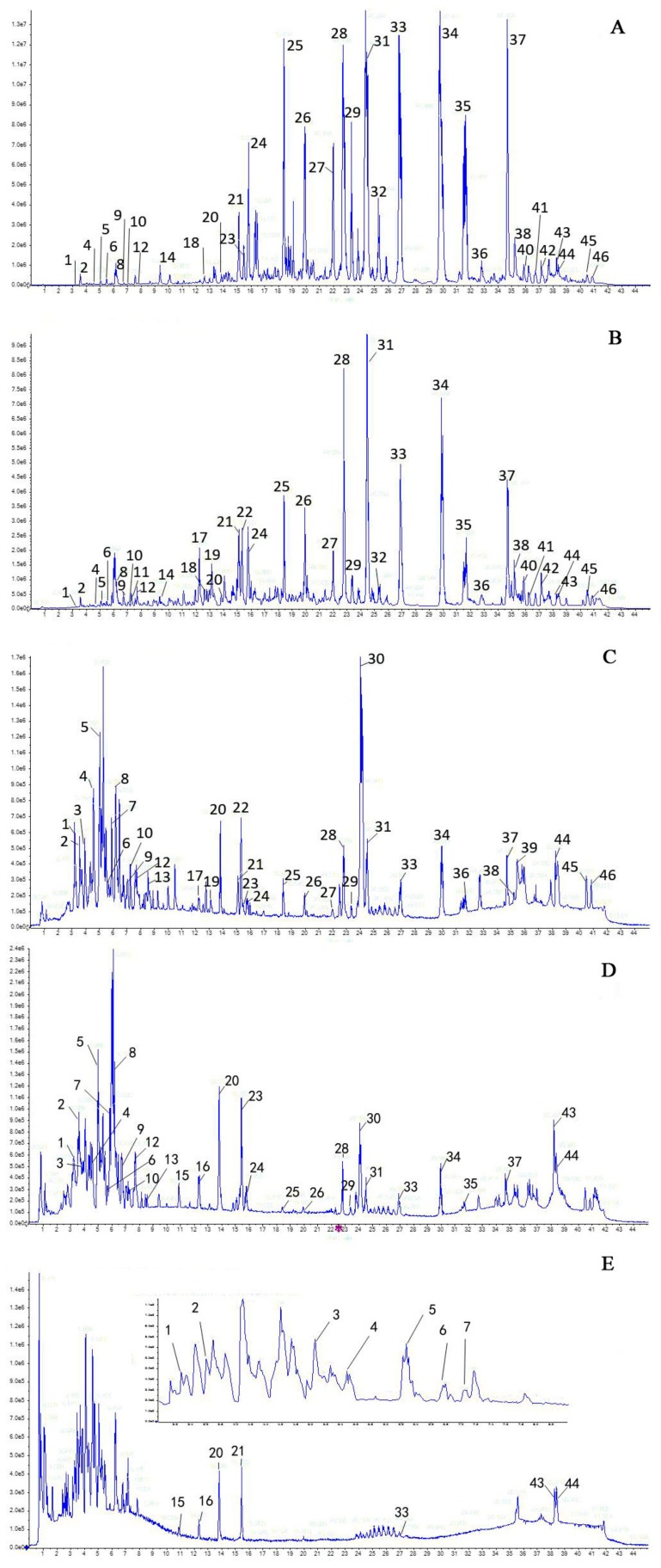
UHPLC fingerprints of different fractions of *Euodiae fructus*: (**A**) petroleum ether fraction; (**B**) chloroform fraction; (**C**) ethyl acetate fraction; (**D**) *n*-butanol fraction; and (**E**) water fraction.

Optimizing the chromatographic and MS conditions, the UHPLC fingerprint of each fraction was successfully developed and a typical chromatogram containing these fractions was presented in Figure 1. On the basis of previous research on UHPLC-Q-TOF-MS [22], ESI-MS collision patterns of major components in *Euodia rutaecarpa* have been studied systematically, and over 80 compounds were identified (relevant work will be reported in another paper). The identification results of the compounds involved in this paper are summarized and described in Table 1.

### 2.2. Hepatotoxicity Induced by Euodia rutaecarpa

Hepatotoxicity induced by *Euodia rutaecarpa* was evaluated by physiological and behavioural changes, histomorphological inspection and the levels of ALT and AST.

#### 2.2.1. Observation of General Condition

The mice in the control group were normal over the tested 7 days (d), and there was nothing out of ordinary regarding their hair colour and diet activity; while the main symptom of mice in the administration group was diarrhoea. They were idle and inactive, as well; their hair colour was gloomy, lacking of a good diet. The weights of all mice slowly increased after administration. In comparison with the control group, high-dose groups of the ethyl acetate, *n*-butanol, and water fractions presented statistical differences 7 days after administration. The influence on the variation of mice weights are recorded and listed in Table 2.

#### 2.2.2. Examination of the Liver-Weight-to-Body-Weight Ratio

The entire doses of different fractions showed increases of the liver-weight-to-body-weight ratio, and all groups had significant differences compared with the control group, except for the low-dose group of petroleum ether, chloroform, and *n*-butanol (*p* < 0.05, *p* < 0.01, summarized in Table 3). In addition, the liver-weight-to-body-weight ratio increased gradually while doses increased, exhibiting a dose-dependent relationship.

#### 2.2.3. Effect on Pathology of Mice Livers

**Figure 2:**
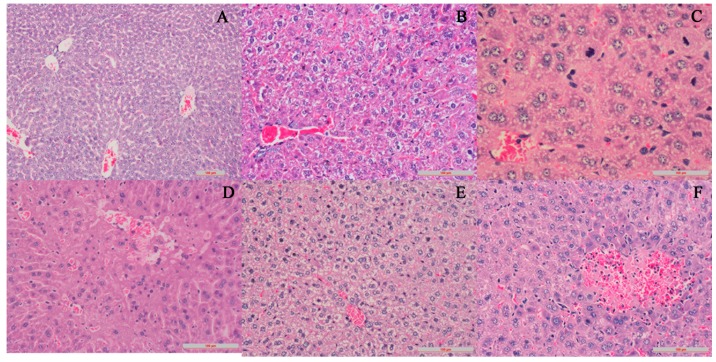
Pathology slices of mice livers (magnification of 200×). (**A**) is the control group; (**B**) is the high dose of petroleum ether group; (**C**) is the high dose of chloroform group; (**D**) is the high dose of butanol group; and (**E**) is the high dose of water group.

In Figure 2A, the structure of hepatic lobule was distinct and liver cells are distributed in a strip shape. The cell nucleus was round and in a vacuole-shape. In Figure 2B, cells in the central-vein area suffered mild edema and congestion. The cytoplasm was bright and transparent. In Figure 2C, liver cells were swelled and the cell boundaries became indistinct. There was spotty necrosis with some cells, of which the nucleus broke down. Some liver cells exhibited regenerative changes: the nucleus was unusually large, chromatin became thicker; the number of nucleoli increased—double, or even triple, the nucleoli could be seen. In Figure 2D, there is piecemeal necrosis in the marginal zone of the hepatic lobule. Infiltration of a small amount of chronic inflammation cells was discovered. In Figure 2E, severe cellular edema can be observed; cytoplasm is bright and transparent showing ballooning degeneration. In Figure 2F, focal necrosis was discovered, and hepatic cords broke down; considerable necroses occurred with haemorrhage and infiltration of chronic inflammation cells.

#### 2.2.4. Effect on Serum Biochemical Indices of Mice

The serum levels of ALT and AST in the control group were within the normal range, while those in the administrated groups rose significantly (*p* < 0.05, *p* < 0.01), results were provided in Table 4. Given that there were no notable etiological differences (behavioural patterns) between control and administrated groups, differences of histomorphological inspection only happened for high-dose groups and there were significant differences for biochemical indices, it would be more sensitive and liable to apply the biochemical index to the spectrum-toxicity relationship analysis.

### 2.3. Spectrum-Toxicity Relationship Analysis

#### 2.3.1. OPLS Results of ALT

OPLS is an improvement to the classical PLS method that offers enhanced model interpretation and is better at finding out what is correlated and uncorrelated with the targeted process. It is often used when attempting to understand the relationship between the raw data and the process; in this case, they are compounds and the corresponding hepatotoxicity caused by those compounds. OPLS employed in this study was performed by the SIMCA software package, providing the results with scatterplots and score plots, which visualized the analytical results. This statistical-based method was more visual-friendly and accurate for screening the hepatotoxic components.

**Figure 3:**
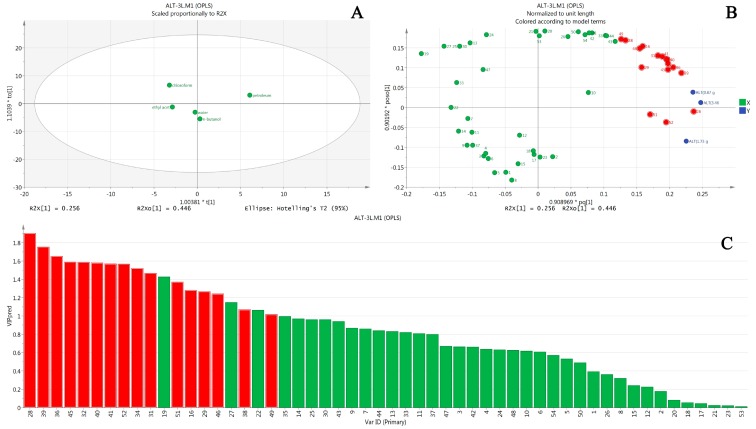
Results of OPLS analysis of AST in hepatotoxicity induced by *Euodia rutaecarpa*. (**A**) is the score scatterplot; (**B**) is the loading scatterplot; and (**C**) is the predictive VIP.

Five fractions of *Euodia rutaecarpa* extract are scattered in different regions in the score scatterplot, as shown in Figure 3A. ALT values are getting larger from left to right along the *x* axis, of which are chloroform, ethyl acetate, water, *n*-butanol, and petroleum ether, respectively. This overview figure illustrated that all the fractions were harmful to mice liver on different levels and, judging from the distance between the petroleum ether fraction and the other four fractions, it is suggested that the petroleum ether fraction damaged livers the most.

Figure 3B is a loading scatterplot, which is used to display the relation between *X*-variables and *Y*-variables. In the current work, it was used to find out which compounds (*X*-variables) correlated to ALT. The *Y*-variable, namely ALT, is on the right of the *y* axis, hence, all the *X*-variables on the right of *y* axis were correlated with the *Y*-variable in a positive manner, while those on the left were otherwise. In addition, the further an *X*-variable is from the origin of the coordinate, the better it is connected to the *Y*-variable. Based on this, dozens of *X*-variables which were all on the right of the *y* axis were preliminarily selected for further screening.

**Figure 4:**
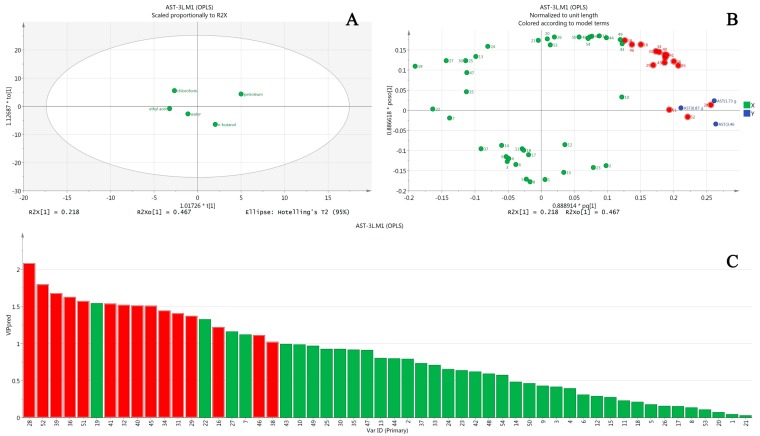
Results of OPLS analysis of AST in hepatotoxicity induced by *Euodia rutaecarpa*. (**A**) is the score scatterplot; (**B**) is the loading scatterplot; and (**C**) is the predictive VIP.

Predictive loading expresses what is correlated to *Y* and how well they are correlated. The VIP (variable importance for the projection) value is the most commonly used method to estimate how much *X*-variables contributed to the correlation with *Y*-variables. Generally, it is considered that a variable with VIP value larger than 1 indicates that it is “important”, meaning it has statistically significance. In Figure 3C, the VIP values are sorted from high to low; consequently, a dozen peaks with VIP values larger than 1 are singled out. Then, taking the loading scatterplot findings into account, which means these variables were supposed to be positively correlated to ALT, the hepatotoxic components induced by *Euodia rutaecarpa* were eventually obtained, which were peaks 28, 39, 36, 45, 32, 40, 41, 52, 34, 31, 51, 16, 29, 46, 38 and 49 respectively. These peaks were coloured entirely in red in both Figure 4B,C to provide better readability.

#### 2.3.2. OPLS Results of AST

The same OPLS method was applied to AST analysis (Figure 4), and a total of 15 compounds were obtained, with peaks 28, 52, 39, 36, 51, 41, 32, 40, 45, 34, 31, 29, 16, 46, and 38. These compounds were probably the major hepatotoxic components as well.

Table 5 summarizes the analysis results of the spectrum-toxicity relationship: 15 compounds affected ALT and AST levels significantly. The compounds marked with “+” in the table were potential hepatotoxic components; while the two marked with “−” were probably liver-protective or toxicity-reductive components. Judging from the biochemical indices and OPLS results, different extract fractions all exhibited hepatotoxicity, with intensities of petroleum ether > *n*-butanol > water > chloroform > ethyl acetate, which is basically consistent with previous reports [10,13,14].

Of the 15 hepatotoxic components, seven were tentatively identified; they were coniferin, 1-methyl-2-undecyl-4(1*H*)-quinolone, 1-methyl-2-[(6*Z*,9*Z*,12*E*)-pentadeca triene]-4(1*H*)-quinolone, evocarpine, 1-methyl-2-[(*Z*)-7-tridecenyl]-4(1*H*)-quinolone, dihydroevocarpine, and 1-methyl-2-tetradecy-4-(1*H*)-quinolone. Of those components, three were reported before [23] and most of the seven components were quinolone alkaloids, suggesting quinolone alkaloids were probably responsible for the hepatotoxicity induced by *Euodia rutaecarpa*. Moreover, it is worth noting that a fact was found interesting that Compound **12**, Rutaevine, limonin, 6-acetoxy-5-epilimonin, and another five compounds were likely to be components that could protect the liver or reduce the hepatotoxicity through OPLS study; however, to verify this hypothesis requires further in-depth study.

## 3. Materials and Methods

### 3.1. Samples, Reagents and Animals

Crude drug samples were collected in Ganchuan town, Xingan County, Jiangxi Province, China, which were later authenticated as *Euodia rutaecarpa* (Juss.) Benth. by Lan Cao, an associate professor in Jiangxi University of TCM (JXUTCM). Voucher specimens are preserved in the Herbarium of Pharmacognosy, School of Pharmaceutical Sciences, JXUTCM. Analytical-grade chloroform, ethyl acetate, and *n*-butanol were all purchased from Xilong Chemical Co., Ltd. (Guangzhou, Guangdong Province, China); ALT and AST kit were purchased from Nanchang Biotech A and C Biotechnical Industry Inc. (Nanchang, Jiangxi Province, China); chromatographic-grade methanol and acetonitrile were purchased from TEDIA (Fairfield, OH, USA); and ultrapure water was prepared by a Milli-Q water purification system (Millipore, Bedford, MA, USA).

SPF-grade Kunming mice with weights ranging from 18 to 22 g, half male and half female, were provided by Hunan SJA Laboratory Animal Co., Ltd. (approval No. SCXK(Xiang)2011-0003. Changsha, Hunan Province, China). The Experimental Animal Ethics Committee of Jiangxi University of TCM approved all animal protocols. The animal experiments were carried out according to the European Community guidelines for the use of experimental animals.

### 3.2. Apparatus and Conditions

The following apparatus and corresponding software were involved in this work: a Nexera X2 UHPLC system (Shimadzu Corporation, Shanghai, China) consisting of a DGU-20A5R degasser, SIL-30AC autosampler, SPD-M20A DAD, CTO-30A oven, and an LC-30AD dual pump; an AB SCIEX Triple TOF 5600+ MS (AB SCEIX, Framingham, MA, USA); the data acquisition software was Analyst TF 1.6 (AB SCEIX, Framingham, MA, USA); the data processing software included Peakview 2.0 (AB SCEIX, Framingham, MA, USA), Masterview 1.0 (AB SCEIX, Framingham, MA, USA), Markerview 1.2.1 (AB SCEIX, Framingham, MA, USA), and SIMCA 14.1 (MKS Umetrics, Umea, Sweden); AU480 automatic biochemical analyser (Beckman Coulter, Shanghai, China); high-speed freezing centrifuge (SIGMA-18, SIGMA Corporation, Shanghai, China); and an electronic analytical balance (AE-240, Perking Sartorius, Beijing, China).

HPLC was performed on a ZORBAX Eclipse plus C18 analytical column (2.1 mm × 100 mm, 1.8 µm). Mobile phases consisted of 0.2% formic acid in both acetonitrile and water using a gradient elution of 5–62% B at 0–20 min, and 65–95% B at 20–40 min. The flow rate was 0.3 mL/min, the injectionvolume was 2 µL, and column oven was 30 °C.

MS detection was achieved by an electrospray ionization source in positive mode with an *m*/*z* scanning range of 100–2000 Da for the MS scan and 50–2000 Da for the TOF scan. GS1 and GS2 were both 60 psi; curtain gas was 35 psi. Ion source temp was set at 500 °C; ISVF was set at 5000 V; DP was 100 V; CE was 10 V for the MS scan and 35 V for the TOF scan; and CES was 15 V.

### 3.3. Preparation of Samples and Sample Solution

The alcohol-water dual extraction method was applied to ensure the samples were extracted completely: accurately weighted sample powder of 2.5 kg was extracted twice by reflux with an eight-fold weight of 75% ethanol for 1.5 and 1 h, then filtrates were combined; the sample residue was decocted twice with a 10-fold weight of water for 1 and 0.5 h, then filtrates were combined. Alcohol extract was concentrated under vacuum until there was no scent of alcohol, then it was combined with water extract and concentrated to acquire the final extract. The final extract was diluted with water, then extracted successively with petroleum ether (60–90 °C), chloroform, ethyl acetate, *n*-butanol, and water for 3–5 times. The corresponding extracts were combined to obtain five extracts from different fractions. Subsequently, the organic solvent was vaporized, after which the extracts were well sealed in vessels and refrigerated in a refrigerator. All extracts were dissolved with the assistance of 5% polysorbate-80 into certain concentrations of solutions for further toxicity study.

Sample solution was prepared as follows: 1 g of the extract that was carefully weighted was dissolved in methanol (with the aid of chloroform) and diluted to exact 25 mL with methanol using a volumetric flask. All solutions were filtered through a 0.22-μm filter membrane for the UHPLC fingerprinting analysis. 

### 3.4. Animal Experiments

One-hundred sixty mice, of which half were male and the other half were female, were randomly divided into 16 groups (10 mice for each), including a control group, a petroleum ether fraction group, a chloroform fraction group, an ethyl acetate fraction group, an *n*-butanol fraction group, and a water group. The administrative doses were calculated according to clinical dose on humans, which were one-fold, two-fold, and four-fold equivalents to the daily administration of the human dose; they were 0.87, 1.73, and 3.46 g/kg for low-dose, intermediate-dose, and high-dose, respectively. A dose of 25 mL/kg was given to all groups, except for the control group, by gavage once every day; the same dose of water was given to the control group instead. The administration of all of the administrated groups lasted for seven consecutive days, during which time weight, eating, drinking, hair color, and other ordinary status were carefully observed.

Blood was collected immediately 60 min after the last administration, then was centrifuged at 3000 r/min for 10 min. Supernatant serum was carefully separated for the measurement of ALT and AST levels using corresponding kits as per the kit instructions.

Mice were sacrificed by cervical dislocation after the blood collection. Livers were excised and weighted for calculating the liver index (shown in Table 6). Certain liver tissue was handled by haematoxylin-eosin staining followed by the inspection for histomorphological changes under an optical microscope.

### 3.5. Data Handling

Presented with $\bar{x}\pm s$, data were handled with SPSS 17.0 statistical software for one-way analysis of variance. Statistical differences were tested between data in different fraction groups and those in the control group. For all tests, differences were considered significant when *p* < 0.05.

The MS raw data acquired were handled by MassLynx V4.1 software, and the preprocessing included peak extract, peak match, peak alignment, peak recognition, and so on. The dataset consisting of sample ID, retention time, *m*/*z* and corresponding ion abundance was obtained after the preprocessing. The dataset and toxicity data were then imported into the SIMCA software package to perform OPLS using UV scaling to study the spectrum-toxicity relationship.

## 4. Conclusions

Based on the findings of the current study, evidence manifested that the “effect” and “toxicity” of *Euodia rutaecarpa* are a unity of opposites, these components cooperate with each other and restrain each other somehow. For instance, evodiamine, rutaecarpin, and evodine are the major components for suppressing pain [27]; limonin can inhibit inflammation and tumors [28]. However, evodiamine, rutaecarpin, and evodine are also cytotoxic to nephrocytes [29]; and rutaecarpin and limonin can result in chromosomal aberration of CHL cells and, hence, are genetoxic [29]. These facts suggest that evodiamine, rutaecarpin, and evodine are both effective and toxic, indicating the effect and the toxicity of TCM go hand in hand, therefore, science-based understanding is undoubtedly in need. 

1-Methyl-2-nonyl-4(1*H*)-quinolone, 1-methyl-2-[(4*Z*,7*Z*)-4,7-tridecadienyl]-4(1*H*)-quinolone, 1-methyl-2-[(6Z,9*Z*,12*E*)-pentadecatriene]-4(1*H*)-quinolone, and 1-methyl-2-pentadecyl-4(1*H*)-quinolone have been reported as ones of the effective components in *Euodia rutaecarpa* [25]; given the findings of this study, whether these components were toxic as well still requires further exploring and researching. If the effective components were the very toxic ones, it would be imperative to define the minimal toxic dose while exerting its maximal effect so that its safety, effect, and reasonability for clinical purpose could be ensured. Nevertheless, if they are different components, how to retain the effective components while removing the toxic ones is a key issue to solve for *Euodia rutaecarpa* toxicity.

## Figures and Tables

**Table 1:** Compounds identified from *Euodia rutaecarpa* extract.

Peak No.	Name	RT (min)	Formula	Ion Mode	Mass (*m*/*z*)	Error (ppm)	Fragment Ions (*m*/*z*)	Ref.
1	Chlorogenic acid	3.27	C_16_H_18_O_9_	[M + H]^+^	355.0483	−2.16	163.0239;145.0175	[23]
4	Hyperoside	4.57	C_21_H_20_O_12_	[M + H]^+^	465.0220	−3.32	303.0072;285.0015	[23,24]
8	Dehydroevodiamine	6.18	C_19_H_15_N_3_O	[M + H]^+^	302.1393	−1.15	286.0555;272.0442	[25]
10	Evodianinine	7.05	C_19_H_13_N_3_O	[M + H]^+^	300.0709	−2.28	285.0506;257.0617	[24]
12	Unknown	7.68	C_20_H_13_NO_4_	[M + H]^+^	332.0890	−2.10	285.0542;257.0657	[5]
14	Ribalinine	9.34	C_15_H_17_NO_3_	[M + H]^+^	260.0643	−1.41	245.0362;227.0352	[5]
16	Coniferin	12.34	C_16_H_22_O_8_	[M + H]^+^	343.0650	−4.12	313.0258;285.0372	[23]
17	Rutaevine	12.23	C_26_H_30_O_9_	[M + H]^+^	487.1184	−0.57	469.1146;443.1410; 425.1333;337.1127	[23,24]
18	Skimmiamine	9.37	C_14_H_13_NO_4_	[M + H]^+^	260.0978	−1.74	204.0474;186.0391	[26]
19	limonin	13.14	C_26_H_30_O_8_	[M + H]^+^	471.1263	−2.03	453.1123;425.1252; 339.1445;161.0445	[24]
21	Evodiamine	15.11	C_19_H_17_N_3_O	[M + H]^+^	304.1078	0.21	171.0781;161.0594; 144.0722;134.0538	[22]
22	6-Acetoxy-5-epilimonin	15.32	C_28_H_32_O_10_	[M + H]^+^	529.1179	−3.14	451.0981;425.1250; 367.0959;161.0451	[23]
23	Trans-caffeoylgluconic acid	15.44	C_16_H_20_O_10_	[M + H]^+^	373.1248	−2.23	358.0505;343.0296; 325.0240	[24]
24	Rutaecarpine	15.78	C_18_H_13_N_3_0	[M + H]^+^	288.1239	0.76	286.0616;271.0537; 244.0585;169.0614	[23,24]
25	1-Methyl-2-nonyl-4(1*H*)-quinolone	18.41	C_19_H_27_NO	[M + H]^+^	296.1802	−1.18	186.0725;173.0667; 158.0471	[24]
26	1-Methyl-2-[(*E*)-1-undecenyl]-4(1*H*)-quinolone	19.93	C_21_H_29_NO	[M + H]^+^	312.1889	−3.54	228.1118;200.0850; 186.0707;173.0652	[24]
27	1-Methyl-2-[(4*Z*,7*Z*)-4,7-tridecadienyl]-4(1*H*)-quinolone	21.99	C_23_H_31_NO	[M + H]^+^	338.2003	−1.60	186.0744;173.0670; 159.0529	[24]
28	1-Methyl-2-undecyl-4(1*H*)-quinolone	22.80	C_21_H_31_NO	[M + H]^+^	314.1985	0.63	186.0701;173.0643; 144.0681;132.0482	[24]
29	1-Methyl-2-[(6*Z*,9*Z*,12*E*)-pentadeca triene]-4(1*H*)-quinolone	23.36	C_25_H_33_NO	[M + H]^+^	364.2089	−1.51	334.1692;308.1591; 268.1355;200.0855; 186.0712;173.0660	[24]
31	Evocarpine	24.50	C_23_H_33_NO	[M + H]^+^	340.2138	−2.75	242.1244;228.1113; 200.0846;186.0703	[24]
32	1-Methyl-2-[(*Z*)-7-tridecenyl]-4(1*H*)-quinolone	25.29	C_23_H_33_NO	[M + H]^+^	340.2136	−3.27	186.0713;173.0665; 159.0531	[24]
33	1-Methyl-2-[(6*Z*,9*Z*)-pentadecadienyl]-4(1*H*)-quinolone	26.89	C_25_H_35_NO	[M + H]^+^	366.2243	−3.91	228.1114;200.0851; 186.0706;173.0652; 159.0535	[24,26]
34	Dihydroevocarpine	29.90	C_23_H_35_NO	[M + H]^+^	342.2411	−0.21	326.2477;298.2107; 186.0745;173.0682	[24,26]
35	1-Methyl-2-[(*Z*)-10-pentadecenyl]-4(1*H*)-quinolone	31.64	C_25_H_37_NO	[M + H]^+^	368.2390	−2.33	326.1982;284.1630; 256.1359;228.1102; 186.0720;173.0665	[24]
36	1-Methyl-2-tetradecy-4-(1*H*)-quinolone	32.79	C_24_H_37_NO	[M + H]^+^	356.2442	−0.87	256.1388;228.1120; 186.0740;173.0680	[24]
37	1-Methyl-2-pentadecyl-4(1*H*)-quinolone	34.68	C_25_H_39_NO	[M + H]^+^	370.2550	−3.45	354.2272;326.1963; 256.1367;200.0849; 186.0718;173.0659	[24]

**Table 2:** Influence of different fractions on body weight of mice ($\bar{x}$ ± s, *n* = 10).

Group	Dose (g/kg)	Before admin.	1 Day after admin.	3 Days after admin.	5 Days after admin.	7 Days after admin.
Control	-	24.5 ± 2.1	25.8 ± 2.3	28.8 ± 2.8	30.2 ± 2.5	32.8 ± 2.7
Petroleum ether fraction	3.46	24.3 ± 1.8	25.7 ± 2.0	28.1 ± 2.1	30.7 ± 2.3	31.7 ± 2.7
	1.73	24.6 ± 1.8	25.2 ± 2.1	28.7 ± 1.9	30.1 ± 1.8	31.4 ± 2.0
	0.87	24.2 ± 1.7	25.6 ± 2.2	29.1 ± 1.6	30.0 ± 1.8	32.2 ± 2.4
Chloroform fraction	3.46	24.3 ± 1.8	25.9 ± 2.0	29.4 ± 1.9	30.2 ± 2.1	31.5 ± 2.4
	1.73	24.5 ± 1.7	25.5 ± 2.3	28.9 ± 2.6	30.8 ± 2.6	30.7 ± 2.5
	0.87	24.7 ± 1.9	25.6 ± 2.1	29.1 ± 2.0	31.2 ± 2.1	30.6 ± 2.4
Ethyl acetate fraction	3.46	24.5 ± 1.7	25.0 ± 2.9	27.7 ± 2.7	29.2 ± 3.0	29.8 ± 2.8 **
	1.73	24.3 ± 1.6	25.9 ± 2.6	27.8 ± 2.8	30.3 ± 2.5	31.0 ± 2.6 *
	0.87	24.7 ± 1.8	25.0 ± 2.8	27.7 ± 2.7	28.8 ± 2.6	29.6 ± 2.5 *
*n*-Butanol fraction	3.46	24.2 ± 1.8	25.1 ± 2.9	27.3 ± 2.7	29.3 ± 2.8	28.1 ± 2.8 **
	1.73	24.4 ± 1.7	25.3 ± 2.9	27.9 ± 2.5	29.5 ± 2.6	30.0 ± 2.9 *
	0.87	24.7 ± 1.8	25.5 ± 1.8	28.1 ± 1.8	29.7 ± 1.82.8	30.1 ± 1.8 *
Water fraction	3.46	24.5 ± 1.7	24.9 ± 2.2	26.9 ± 2.8	28.9 ± 2.9	30.0 ± 2.7 *
	1.73	24.8 ± 1.8	25.0 ± 2.7	27.0 ± 2.9	29.2 ± 2.8	30.8 ± 2.6
	0.87	24.4 ± 1.6	25.3 ± 2.6	27.1 ± 2.9	29.1 ± 2.8	30.5 ± 2.9

Note: * *p* < 0.05, ** *p* < 0.01 compared with control group.

**Table 3:** The influence of different fractions on the liver-weight-to-body-weight ratio ($\bar{x}$ ± s, *n* = 100).

Group	Dose (g/kg)	Liver-Weight-to-Body-Weight Ratio (g/100 g)
Control	-	4.297 ± 0.489
Petroleum ether fraction	3.46	4.625 ± 0.784 **
	1.73	4.529 ± 0.829 *
	0.87	4.378 ± 0.596
Chloroform fraction	3.46	4.704 ± 0.160 **
	1.73	4.652 ± 0.658 **
	0.87	4.508 ± 0.784
Ethyl acetate fraction	3.46	5.155 ± 0.672 **
	1.73	5.054 ± 0.830 **
	0.87	4.925 ± 0.668 *
*n*-Butanol fraction	3.46	5.407 ± 0.835 **
	1.73	4.950 ± 0.851 *
	0.87	4.790 ± 0.850
Water fraction	3.46	5.362 ± 0.423 **
	1.73	5.044 ± 0.782 *
	0.87	4.828 ± 0.834 *

Note: * *p* < 0.05, ** *p* < 0.01 compared with control group.

**Table 4:** Influence of different fractions on serum biochemical indices of mice ($\bar{x}$ ± s, *n* = 10).

Group	Dose (g/kg)	AST (IU/L)	ALT (IU/L)
Control	-	98.26 ± 12.97	48.32 ± 5.52
Petroleum ether fraction	3.46	156.12 ± 16.47 **	94.78 ± 20.87 **
	1.73	150.12 ± 27.10 **	81.22 ± 16.51 **
	0.87	122.58 ± 30.43 *	78.02 ± 12.82 **
Chloroform fraction	3.46	130.87 ± 18.84 **	71.49 ± 19.74 **
	1.73	124.39 ± 29.30 *	65.54 ± 9.08 **
	0.87	121.67 ± 21.64 *	63.17 ± 7.14 **
Ethyl acetate fraction	3.46	126.06 ± 20.22 **	68.65 ± 11.28 **
	1.73	119.57 ± 18.68 *	66.92 ± 12.00 **
	0.87	113.69 ± 16.75 *	61.04 ± 14.51 *
*n*-Butanol fraction	3.46	151.04 ± 30.86 **	79.18 ± 18.62 **
	1.73	136.05 ± 22.43 **	77.72 ± 37.01 **
	0.87	125.69 ± 34.91 *	67.66 ± 11.30 *
Water fraction	3.46	142.41 ± 21.83 **	78.64 ± 15.38 **
	1.73	130.04 ± 35.56 *	77.82 ± 12.70 **
	0.87	115.02 ± 19.11 *	62.87 ± 16.56 *

Note: * *p* < 0.05, ** *p* < 0.01 compared with control group.

**Table 5:** Results of the OPLS analysis of hepatotoxicity induced by *Euodia rutaecarpa*.

	Peak No.	16	19	22	28	29	31	32	34	36	38	39	40	41	45	46	49	51	52
Group																			
ALT		+	−	−	+	+	+	+	+	+	+	+	+	+	+	+	+	+	+
AST		+	−	−	+	+	+	+	+	+	+	+	+	+	+	+	/	+	+
ALT + AST		+	−	−	+	+	+	+	+	+	+	+	+	+	+	+	/	+	+

Note: “+” stands for positive correlation, “−” stands for negative correlation, “/” stands for no significant correlation. The assignments of peaks are in Table 1.

**Table 6:** The influence of different fractions on liver viscera indices in mice ($\bar{x}$ ± s, *n* = 10).

Group	Dose (g/kg)	Liver/Body (g/100 g)
Control	-	4.297 ± 0.489
Petroleum ether fraction	3.46	4.625 ± 0.784 **
	1.73	4.529 ± 0.829 *
	0.87	4.378 ± 0.596
Chloroform fraction	3.46	4.704 ± 0.160 **
	1.73	4.652 ± 0.658 **
	0.87	4.508 ± 0.784
Ethyl acetate fraction	3.46	5.155 ± 0.672 **
	1.73	5.054 ± 0.830 **
	0.87	4.925 ± 0.668 *
*n*-Butanol fraction	3.46	5.407 ± 0.835 **
	1.73	4.950 ± 0.851 *
	0.87	4.790 ± 0.850
Water fraction	3.46	5.362 ± 0.423 **
	1.73	5.044 ± 0.782 *
	0.87	4.828 ± 0.834 *

Note: * *p* < 0.05, ** *p* < 0.01 compared with control group.
